# Early restriction of placental growth results in placental structural and gene expression changes in late gestation independent of fetal hypoxemia

**DOI:** 10.14814/phy2.13049

**Published:** 2016-12-06

**Authors:** Song Zhang, Paige Barker, Kimberley J. Botting, Claire T. Roberts, Christine M. McMillan, Isabella Caroline McMillen, Janna L. Morrison

**Affiliations:** ^1^ Early Origins of Adult Health Research GroupSansom Institute for Health ResearchUniversity of South AustraliaAdelaideSouth AustraliaAustralia; ^2^ The Robinson Research Institute and Adelaide Medical SchoolUniversity of AdelaideAdelaideSouth AustraliaAustralia

**Keywords:** Carunclectomy, fetus, hypoxemia, intrauterine growth restriction, placenta, pregnancy

## Abstract

Placental restriction and insufficiency are associated with altered patterns of placental growth, morphology, substrate transport capacity, growth factor expression, and glucocorticoid exposure. We have used a pregnant sheep model in which the intrauterine environment has been perturbed by uterine carunclectomy (Cx). This procedure results in early restriction of placental growth and either the development of chronic fetal hypoxemia (PaO_2≤_17 mmHg) in late gestation or in compensatory placental growth and the maintenance of fetal normoxemia (PaO_2_>17 mmHg). Based on fetal PaO_2_, Cx, and Control ewes were assigned to either a normoxemic fetal group (Nx) or a hypoxemic fetal group (Hx) in late gestation, resulting in 4 groups. Cx resulted in a decrease in the volumes of fetal and maternal connective tissues in the placenta and increased placental mRNA expression of *IGF2,* vascular endothelial growth factor (*VEGF*), *VEGFR‐2*,*ANGPT2,* and *TIE2*. There were reduced volumes of trophoblast, maternal epithelium, and maternal connective tissues in the placenta and a decrease in placental *GLUT1* and *11βHSD2* mRNA expression in the Hx compared to Nx groups. Our data show that early restriction of placental growth has effects on morphological and functional characteristics of the placenta in late gestation, independent of whether the fetus becomes hypoxemic. Similarly, there is a distinct set of placental changes that are only present in fetuses that were hypoxemic in late gestation, independent of whether Cx occurred. Thus, we provide further understanding of the different placental cellular and molecular mechanisms that are present in early placental restriction and in the emergence of later placental insufficiency.

## Introduction

In mammals, the placenta is the primary interface between the mother and fetus and plays an essential role in maintaining fetal growth and development by facilitating the transfer of oxygen and nutrients to the fetus and removing fetal carbon dioxide and wastes. Failure of the placenta to deliver an adequate supply of substrates to the fetus is termed placental insufficiency and results in intrauterine growth restriction (IUGR). IUGR is clinically defined as a birth weight below the tenth centile for gestational age, that is, where the fetus does not meet its growth potential (McMillen et al. 2001; Morrison 2008). IUGR affects 5–10% of pregnancies in developed countries (Limesand et al. 2006; Bamfo and Odibo 2011; Li et al. 2012), and is associated with a high incidence of perinatal morbidity and mortality, as well as an increased risk of cardiovascular disease, hypertension and type 2 diabetes in adulthood (Barker et al. 1993, 2010; McMillen and Robinson 2005).

A range of human and animal studies in IUGR pregnancies have suggested that placental restriction and insufficiency may be associated with a series of morphological and functional changes in the placenta, such as altered growth, substrate transport capacity, increased apoptosis, and autophagy, as well as increased glucocorticoid exposure (Zhang et al. 2015). Previous reports have shown a decrease in the surface area, volume, and number of terminal villi and capillaries in placentas from IUGR compared with normal human pregnancies (Krebs et al. 1996; Mayhew et al. 2003). Placental expression of vascular endothelial growth factor (VEGF), basic fibroblast growth factor (FGF2), angiopoietin‐1 (ANGPT1), angiopoietin‐2 (ANGPT2), and angiopoietin receptor (TIE‐2) are significantly higher in human IUGR pregnancies and in the hyperthermia‐induced sheep model of IUGR (Regnault et al. 2002, 2003; Hagen et al. 2005; Barut et al. 2010). Placental insulin‐like growth factor 2 (*IGF2*) and *IGF1* mRNA expression were also increased at 55 and 90 days of gestation in hyperthermia‐induced IUGR pregnancies (de Vrijer et al. 2006). In humans, placental glucose transporter 1 (GLUT1) mRNA expression and protein abundance was decreased in the presence of an IUGR fetus, suggesting placental glucose transport capacity is impaired in these pregnancies (Dubova et al. 2013; Janzen et al. 2013). Placental 11*β*‐hydroxysteroid dehydrogenase 2 (11*β*HSD2) expression and activity was significantly reduced in deliveries complicated by IUGR compared with term or appropriately grown preterm deliveries, suggesting that increased intraplacental glucocorticoids may contribute to reduced substrate delivery in the IUGR pregnancy (Shams et al. 1998).

An experimental model of placental restriction has been developed in the sheep, using uterine carunclectomy (Cx), a procedure involving surgical removal of most endometrial caruncles from the uterus of nonpregnant ewes prior to conception. During a subsequent pregnancy, fewer placentomes form, resulting in the restriction of fetal substrate supply, chronic hypoxemia and fetal growth restriction throughout late gestation (Alexander 1964; McMillen et al. 2001; Morrison 2008). This results in an increase in the volume density of the trophoblast and the feto‐maternal syncytium within the placentomes (Robinson et al. 1995); however, no study has investigated the molecular changes that occur in the placenta after this intervention. One important feature of carunclectomy is that it does not inevitably lead to chronic fetal hypoxemia in late gestation because there is a degree of compensatory placental growth or efficiency that may be sufficient to result in the maintenance of fetal normoxemia (Butler et al. 2002). Therefore, in this model, it is possible to determine whether Cx induces early restriction of placental growth, differentiation and function, independently of whether chronic fetal hypoxemia develops in late gestation. It is also possible to determine whether placental growth and function is altered in fetuses that were hypoxemic in late gestation, independently of whether Cx occurred. In sheep, the type A and B placentomes predominate throughout gestation and account for 60% or more of the total number in a normal pregnancy (Ward et al. 2006). The gross morphology of placentomes also changes progressively throughout gestation with an increase in the number of everted (type C and D) placentomes in late gestation (Alexander 1964; Fowden et al. 2006). Thus, type A and B placentomes may be more likely to show any differences in histology and gene expression specific to early restriction of placental growth induced by Cx, and subsequent chronic fetal hypoxemia in late gestation. In this study, we have determined the impact of Cx associated with either fetal hypoxemia (Hx) or normoxemia (Nx) on placental growth, transport capacity, apoptosis, autophagy, and glucocorticoid exposure.

## Methods

### Ethical approval

All experimental procedures were approved by the University of Adelaide and the IMVS/University of South Australia Animal Ethics Committee and performed according to the guidelines of the Australian code of practice for the care and use of animals for scientific purposes.

### Animals and surgery

#### Carunclectomy surgery

Forty nonpregnant Merino ewes were randomly assigned to surgical removal of the majority of the endometrial caruncles (leaving four visible caruncles in each horn), as previously described (carunclectomy; Cx (Danielson et al. 2005; Edwards et al. 1999)) with general anesthesia (induced, intravenous injection of sodium thiopentone (1.25 g Pentothal, Rhone Meriux, Pinkenba, QLD, Australia); maintained, 2% isoflurane in O_2_). After a minimum 10‐week recovery period, these ewes and 38 Control (no previous surgery) ewes entered a mating program. Pregnancy in both Control and Cx ewes was confirmed by ultrasound at ~55 days gestation (term, 150 ± 3 days). Only fetuses from a singleton pregnancy were included in the study.

#### Fetal catheterization surgery

At 115–125 days gestation, both Control and Cx ewes were anesthetized as described above and catheters were implanted in the maternal jugular vein, fetal jugular vein, fetal carotid artery, and amniotic cavity (Edwards et al. 1999; Danielson et al. 2005). Fetal catheters were exteriorized through a small incision in the ewes’ flank. At surgery, antibiotics were administered intramuscularly to the ewe (153.5 mg procaine penicillin, 393 mg benzathine penicillin; 500 mg dihydrostreptomycin, Lyppards, SA, Australia) and fetus (150 mg procaine penicillin, 112.5 mg benzathine penicillin; 250 mg dihydrostreptomycin, Lyppards, SA, Australia). Ewes were housed in individual pens in animal holding rooms with a 12 h light–dark cycle. After surgery, antibiotics were administered intramuscularly to each ewe for 3 day and to each fetus intra‐amniotically (500 mg ampicillin, Lyppards, South Australia, Australia) for 4 day.

### Arterial blood gas measurements

Fetal arterial blood samples (0.5 mL) were collected daily for the measurement of PaO_2_, PaCO_2,_ pH, hemoglobin (Hb), hematocrit, base excess, and oxygen saturation (SaO_2_), using an ABL analyser (Radiometer, Copenhagen, Denmark) with the temperature corrected to 39°C. Oxygen content was calculated using the formula ((PaO_2_ × 0.003) + [Hb] × (SO_2_/100) × 1.39) (Danielson et al. 2005). Mean gestational blood gas values were calculated as the average of all fetal blood gas values collected from three days postsurgery until postmortem. Chronic hypoxemia was defined as a mean gestational PaO_2_ of less than or equal to 17 mmHg (Butler et al. 2002; Danielson et al. 2005; Morrison et al. 2007; Dyer et al. 2009). Our previous studies have shown that there is a continuous range of PaO_2_ and fetal body weights in normally grown and Cx fetuses, and that when the mean gestational PaO_2_ is less than 17 mmHg, the fetal body weight is <10th centile (Danielson et al. 2005; Morrison et al. 2007; Dyer et al. 2009; Wang et al. 2011). Control and Cx ewes were allocated to either a Nx group (Nx; arterial PaO_2_ > 17 mmHg) or a Hx group (Hx; PaO_2_ _≤_ 17 mmHg), resulting in four treatment groups (Control+Nx, *n* = 29; Control+Hx, *n* = 9; Cx+Nx, *n* = 14; Cx+Hx, *n* = 26).

### Postmortem and tissue collection

Ewes (Control+Nx, *n* = 29; Control+Hx, *n* = 9; Cx+Nx, *n* = 14; Cx+Hx, *n* = 26) were humanely killed between 130 and 134 days gestation with an overdose of sodium pentobaritone (Lethobarb; 25 mL; 325 mg/mL, Virbac Aus, Peakhurst, NSW, Australia) via the maternal jugular vein catheter. Fetuses were delivered by hysterectomy and weighed. Placentomes were removed from the uterus and total placentome number, type, and weight of each placentome was recorded (Vatnick et al. 1991; Ward et al. 2006). Placental efficiency was calculated, using the equation fetal weight divided by placental weight (Regnault et al. 2003; Poudel et al. 2015). Placentomes were either frozen in liquid nitrogen for molecular analyses and/or fixed with 4% paraformaldehyde and embedded in paraffin for the morphological analyses.

### Quantification of mRNA expression, using real‐time RT‐PCR

All essential information regarding our procedure is included as per the MIQE guidelines (Bustin et al. 2009). Total RNA was extracted from the inverted (type A or B) placentomes (Control+Nx, *n* = 20; Control+Hx, *n* = 8; Cx+Nx, *n* = 11; Cx+Hx, *n* = 16), using the miRNeasy kit following the manufacturer's protocol (Qiagen, Chadstone, VIC, Australia). The ratio of the optical density at 260and 280 nm was used to calculate the correct dilutions of extracted RNA used for cDNA synthesis. cDNA was synthesized by reverse transcription, using Superscript III (Invitrogen by Life Technologies, Mulgrave, VIC, Australia). A no template control (NTC) containing no RNA transcript and a no amplification control (NAC) containing no Superscript III were used to check for reagent contamination and genomic DNA contamination, respectively. The relative expression of mRNA transcripts in the placentome was measured by quantitative real‐time PCR, using the ViiA™ 7 Real‐Time PCR System (Applied Biosystems by Life Technologies, Mulgrave, VIC, Australia). A PCR reaction consisted of Fast SYBR Green (Sigma, Australia), forward and reverse primers (GeneWorks, Adelaide, SA, Australia), molecular‐grade H_2_O, and cDNA (Table 1). The most stable three reference genes, including phosphoglycerate kinase 1, *β*‐actin, and glyceraldehyde‐3‐phosphate dehydrogenase were selected from a panel of candidate genes based in the geNorm component of qbaseplus 2.0 software (Biogazelle, Zwijnaarde, Belgium) (Passmore et al. 2009; Soo et al. 2012; Botting et al. 2014). The abundance of each transcript was normalized to three reference genes and expressed as mean normalized expression. Target genes of interest fell into several categories and primer sequences were validated for use in the sheep in this study or in prior studies, including hypoxia‐inducible factors (*HIF1A*,* HIF1B*,* HIF2A*, and *HIF3A* (Botting et al. 2014)), vasculogenesis and angiogenesis (*VEGF*, VEGF receptors including *VEGFR‐1* and *VEGFR‐2*,* FGF2*,* ANGPT1*,* ANGPT2*,* TIE2* (Botting et al. 2014); Table 1), glucose transporters (*GLUT1*,* GLUT3*, and *GLUT4* (Botting et al. 2014; Muhlhausler et al. 2009)), amino acid transporters (cationic amino acid transporter (*CAT‐1*), large neutral amino acid transporter (*LAT‐1*), sodium‐coupled neutral amino acid transporters (*SNAT1* and *SNAT4*); Table 1), fatty acid transporters, and binding proteins (*FATP1*,* FATP4*,* FABP5*, and fatty acid translocase (*CD36*) (Wang et al. 2013; Lie et al. 2014); Table 1), IGFs and receptors (*IGF1*,* IGF2*,* IGF1R* and *IGF2R* (MacLaughlin et al. 2007; Zhang et al. 2010)), apoptosis (BCL‐2‐like protein 4 (*BAX*), B‐cell lymphoma 2 (*BCL‐2*), and *p53* (Botting et al. 2014); Table 1), autophagy (*BECLIN‐1*, light chain three isoform B (*LC3B*), and lysosomal‐associated membrane protein 1 (*LAMP1*) (Botting et al. 2014)), and glucocorticoid action (*11βHSD1*,* 11βHSD2*, and glucocorticoid receptor (*GR*) (Zhang et al. 2010; McGillick et al. 2014)).

**Table 1:** Primer sequences for real time RT‐PCR

Gene	Accession number	Sequence
*VEGFR‐2*	AF513909.1	*Fwd: 5′‐* TTGATTGCTGGCATGGGGAT ‐3′*Rev: 5′‐* AGGCAGAGAGAGTCCCGAAT ‐3′
*FGF2*	NM_001001855.2	*Fwd: 5′‐* GTGCAAACCGTTACCTTGCT ‐3′*Rev: 5′‐* ACTGCCCAGTTCGTTTCAGT ‐3′
*CAT‐1*	AF212146	*Fwd: 5′‐* CAGACGGGCTTTTTACCGGA ‐3′*Rev: 5′‐* CAACTCCCCTTTCACCAGGG ‐3′
*LAT‐1*	AY162432	*Fwd: 5′‐* CGTCAATGGGTCCCTCTTCA ‐3′*Rev: 5′‐* AAGGCGTAAAGCAGGGTCAT ‐3′
*SNAT1*	XM_004006423.1	*Fwd: 5′‐* GAGTCGTTGGCGTTACATCT ‐3′*Rev: 5′‐* ATTCGCTGAGTTCCCTTATCC ‐3′
*SNAT4*	XM_004006419	*Fwd: 5′‐* CATGGCAGTGGAGTGGAGTT ‐3′*Rev: 5′‐* TAGGAAGGACCTCAGGGTGG ‐3′
*FATP4*	XM_004005604.1	*Fwd: 5′‐* CGTGGTGCATAGCAGGTATTA ‐3′*Rev: 5′‐* GTTTCCTGCAGAGTGGTAGAG ‐3′
*FABP5*	NM_001145180.1	*Fwd: 5′‐* ATCAGGAATGGGATGGAAAGG ‐3′*Rev: 5′‐* AGACCCGAGTACAGGTAACA ‐3′
*p53*	NM_001009403.1	*Fwd: 5′‐* GCTATGGGTCGACTCGCCGC ‐3′*Rev: 5′‐* GGGGACTGCGCCTCACAACC ‐3′

### Placental histology and morphometric study

The inverted placentomes (Control+Nx, *n* = 5; Control+Hx, *n* = 5; Cx+Nx, *n* = 3; Cx+Hx, *n* = 3) were cut (5 *μ*m) and stained with Masson's trichrome, using published methods (Roberts et al. 2001; MacLaughlin et al. 2005; Fletcher et al. 2007). Sections were examined with a 20× objective lens and a 10× ocular lens on an Olympus VANOX‐AHT microscope (Olympus Optical Co., Ltd., Tokyo, Japan), using a Colorview I camera with AnalySIS image analysis software (Soft Imaging Systems, Gulfview Heights, SA, Australia). The proportions of placental trophoblast, fetal capillaries, fetal connective tissue, maternal epithelium, maternal capillaries, and maternal connective tissue were quantified, using point counting with an isotropic L‐36 Merz transparent grid placed on the monitor screen. A random systematic field selection method was utilized. Ten fields (360 points) were counted in each section, 1 mm apart, with the aid of the stage micrometer. The volume density (***V***_d_) of each of the specified components of the placentome and placenta was calculated, using the following formula, *V*_d_ = *P*_a_/*P*_T_, where *P*_a_ is the total number of points falling on a particular component and *P*_T_ is the total number of points in the section (Roberts et al. 2001; MacLaughlin et al. 2005; Fletcher et al. 2007). The estimated volume of each of the specified components in each individual placentome or placenta was calculated by multiplying the volume density of each component by the weight of the individual placentome or total placenta.

Intercept counting on the same grid on the same fields was utilized to calculate the surface density (*S*_v_) of trophoblast, taking into account the total magnification on the monitor screen,using the formula, *S*_v_ = 2 × *I*_a_/*L*_T_, where *I*_a_ is the number of intercepts with the line and *L*_T_ is the total length of the lines applied (Roberts et al. 2001; MacLaughlin et al. 2005; Fletcher et al. 2007). Total surface area for the placentome from which the section was cut and total placenta was estimated by multiplying the surface density by placentome weight or total placental weight and assumes that surface density is similar in all placentomes (Roberts et al. 2001; MacLaughlin et al. 2005; Fletcher et al. 2007). The arithmetic mean barrier of trophoblast to diffusion was calculated using the formula, barrier thickness, *B*_T_=*V*_d_/*S*_v_, where *V*_d_ is the volume density of trophoblast and ***S***_v_ is the surface density of trophoblast (Roberts et al. 2001; MacLaughlin et al. 2005; Fletcher et al. 2007).

### Statistical analysis

All data are presented as mean ± standard error of the mean (SEM). The effects of treatment (Control vs. Cx) representing early placental growth from conception, fetal hypoxemia (Nx vs Hx) representing late placental insufficiency and fetal sex were examined by a 3‐way ANOVA on blood gas measurements, fetal weight, relative brain weight, total placental weight, number of placentomes, mean placentome weight, placental efficiency as well as placental mRNA expression, using IBM Statistical Package for Social Scientists Statistics version 21 (SPSS Inc., Chicago, IL). The effects of treatment and hypoxemia were determined by a 2‐way ANOVA on the placental morphology data, as the sample size was too small to investigate the effect of sex in this part of the data. To determine the effect of placentome type, the placentome data was analyzed, using STATA12 (Data analysis and statistical software for repeated measures [StataCorp LP, College Station, TX]) for a 3‐way ANOVA (with the factors including treatment, hypoxemia and placentome type (inverted (A and B) or everted (C and D)) with animal number nested within treatment groups to identify placentomes coming from the same animal. When a significant interaction between major factors was identified, the data were split on the basis of the interacting factors and re‐analyzed. Linear regression and correlation analyses were performed, using Sigma Plot 12.5 Software (Systat Software Inc, San Jose, CA). A probability level of 5% (*P *<* *0.05) was considered to be statistically significant.

## Results

### Fetal arterial blood gas information

Mean gestational PaO_2_ was lower (*P *<* *0.05), while PaCO_2_, hemoglobin and hematocrit were higher (*P *<* *0.05) in the Hx groups compared with the Nx groups, regardless of whether they were Control or Cx animals (Table 2). There was no effect of Cx, Hx, or an interaction between the effects of Cx and Hx on mean gestational pH and base excess (Table 2). Mean gestational oxygen saturation and oxygen content were lower (*P *<* *0.05) in the Cx compared with the Control groups, and were also lower (*P *<* *0.05) in the Hx compared with the Nx groups. There was no effect of fetal sex on any of the fetal arterial blood gas parameters.

**Table 2:** Fetal mean gestational arterial blood gas measurements

	Control		Cx	
	Nx(*n* = 29)	Hx(*n* = 9)	Nx(*n* = 14)	Hx(*n* = 26)
PaO_2_ (mmHg)	21.6 ± 0.5	15.0 ± 0.5^a^	19.7 ± 0.5	14.4 ± 0.4^a^
PaCO_2_ (mmHg)	47.9 ± 0.7	52.2 ± 1.5^a^	49.3 ± 0.9	49.8 ± 0.8^a^
pH	7.373 ± 0.005	7.359 ± 0.007	7.367 ± 0.007	7.363 ± 0.004
Hemoglobin (g/dL)	9.0 ± 0.2	10.9 ± 0.6^a^	8.8 ± 0.4	10.7 ± 0.4^a^
Hematocrit (%)	27.6 ± 0.7	35.4 ± 2.1^a^	28.2 ± 1.4	34.6 ± 1.6^a^
O_2_ saturation (%)	66.6 ± 1.3	45.3 ± 3.5^a^	58.2 ± 2.2^b^	37.8 ± 2.2^b^^,^ a
Base excess (mEq/L)	2.0 ± 0.5	3.0 ± 0.8	2.0 ± 0.6	2.1 ± 0.4
O_2_ content (mL/dL)	8.4 ± 0.2	6.8 ± 0.4^a^	7.2 ± 0.3^b^	5.6 ± 0.3^b^^,^ a

Cx, carunclectomy. Nx, normoxemic group. Hx, hypoxemic group. Values are mean ± SEM.

a Denotes an effect of hypoxemia (Nx vs. Hx)

b Denotes an effect of Cx treatment (Control vs. Cx). *P *<* *0.05.

### Effects of carunclectomy and hypoxemia on fetal weight, brain weight and placental weight

**Figure 1:**
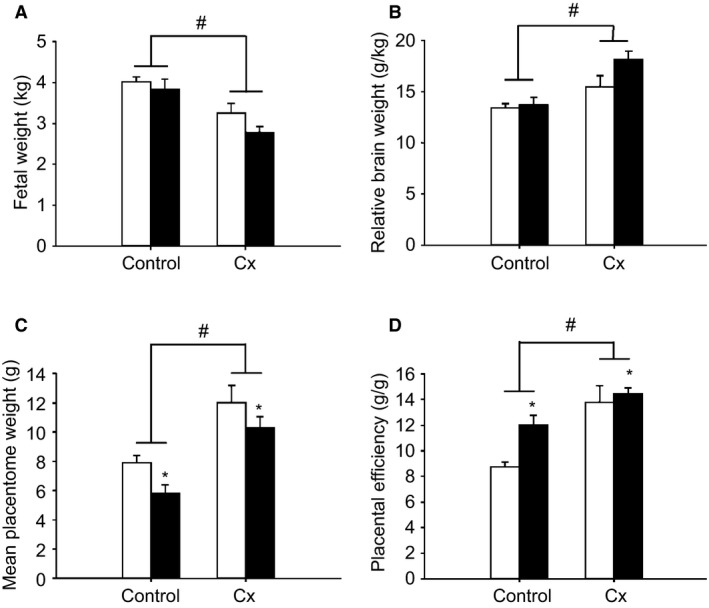
The effects of carunclectomy and hypoxemia on fetal weight (A), relative brain weight (B), mean placentome weight (C) and placental efficiency (D) in the Control+Nx, Control+Hx, Cx+Nx, and Cx+Hx groups. Nx, Normoxemic group, open bars; Hx, Hypoxemic group, closed bars; *, denotes an effect of hypoxemia (Nx vs. Hx); ^#^, denotes an effect of Cx treatment (Control vs. Cx); *P *<* *0.05.

Fetal weight was lower (*P *<* *0.05) in the Cx groups (26% reduction) compared with the Control groups and this occurred independently of whether the fetus was Nx or Hx (Fig. 1). Fetal weight was higher in male (3.60 ± 0.14 kg) than in female fetuses (3.25 ± 0.15 kg; *P *<* *0.05) in both the Control and Cx groups. Relative brain weight was higher (*P *<* *0.05) in the Cx groups compared with the Control groups (Fig. 1), independent of fetal sex and Hx.

**Figure 2:**
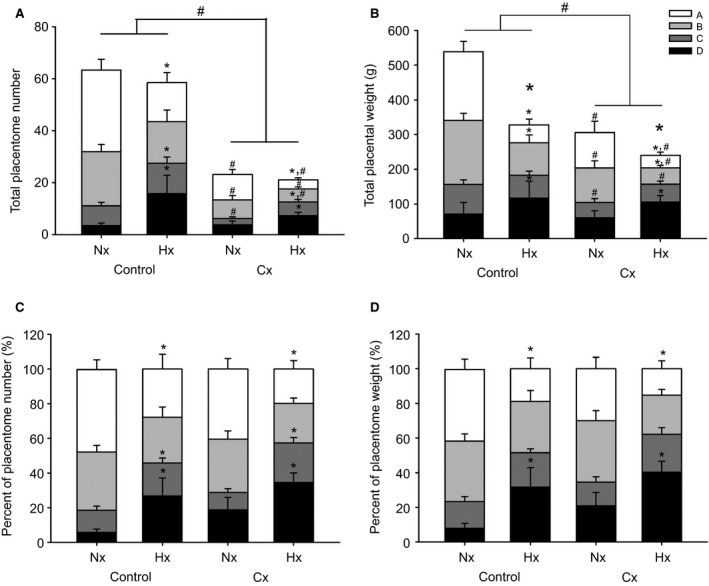
The effects of carunclectomy and hypoxemia on total placentome number (A), total placentome weight (B), percent of placentome number (%; C) and percent of placentome weight (%; D) among A‐, B‐, C‐, and D‐ placentome types. *, denotes an effect of hypoxemia (Nx vs. Hx); #, denotes an effect of Cx treatment (Control vs. Cx); *P *<* *0.05.

**Figure 3:**
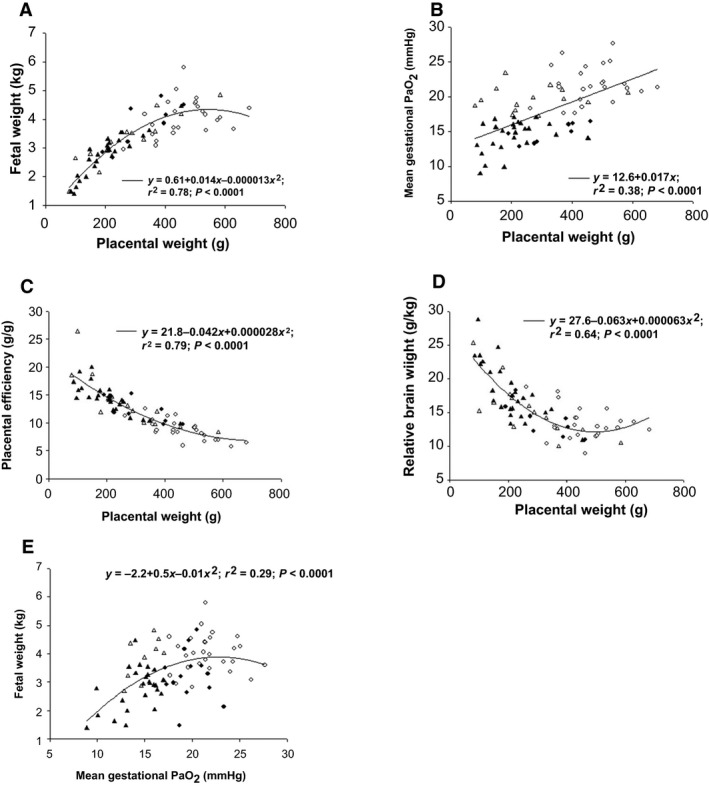
The relationships between placental weight and fetal weight (A), mean gestational PaO_2_ (B), placental efficiency (C) and relative brain weight (D), as well as the relationship between mean gestational PaO_2_ and fetal weight (E) in the Control+Nx (○), Control+Hx (Δ), Cx+Nx (●) and Cx+Hx (▲) groups. *P *<* *0.0001.

Total placental weight was lower in the Cx compared with the Control groups (*P *<* *0.05), and also lower in the Hx compared with the Nx groups (*P *<* *0.05; Fig. 2), irrespective of the sex of the fetus. Total placentome number was lower in the Cx compared with the Control groups independent of whether the fetus was Nx or Hx (*P *<* *0.05; Fig. 2), and was higher in male (41.7 ± 4.0) compared to female fetuses (37.4 ± 3.8; *P *<* *0.05). Mean placentome weight was higher (*P *<* *0.05) in the Cx compared with the Control groups, and was also lower (*P *<* *0.05) in the Hx compared with the Nx groups (Fig. 1). Placental efficiency was higher in the Cx compared with the Control groups and was also higher in the Hx compared with the Nx groups, irrespective of the sex of the fetus (*P *<* *0.05; Fig. 1). There were significant relationships between placental weight and fetal weight, between placental weight and mean gestational PaO_2_, between placental weight and placental efficiency as well as between placental weight and relative brain weight when all treatment groups were considered (Fig. 3). There was also a significant relationship between mean gestational PaO_2_ and fetal weight when all treatment groups were considered (Fig. 3).

There was an interaction between the effects of Cx, Hx and placentome type on placentome numbers, placentome weights and percentages of placentome numbers and weights among type A, B, C and D placentomes (*P *<* *0.05; Fig. 2). The effects of Cx and Hx were then examined in individual types of placentomes. The total numbers of type A, B and C placentomes were significantly lower in the Cx compared with the Control groups. The total number of type A placentomes was lower but the total numbers of type C and D placentomes were higher in the Hx compared with the Nx groups. The total weights of type A, B and C placentomes were lower in the Cx compared with the Control groups. The total weights of type A and B placentomes were lower but the total weight of type D placentomes was higher in the Hx compared with the Nx groups. There was a lower percentage of type A placentome numbers, but higher percentages of type C and D placentome numbers in the Hx compared with the Nx groups. Similarly, there was a lower percentage of type A placentomes by weight, but higher percentage of type D placentomes by weight in the Hx compared with the Nx groups.

### Effects of carunclectomy and hypoxemia on mid‐saggital placental morphology

There was no effect of Cx or Hx on volume density of trophoblast, fetal capillaries, fetal connective tissue, maternal epithelium, maternal capillaries and maternal connective tissue (Table 3). The absolute volumes of trophoblasts and maternal capillaries in the placentome were higher (*P *<* *0.05) in the Cx compared with the Control groups, regardless of whether the fetus was Nx or Hx (Table 3). There was no effect of Cx or Hx on the absolute volumes of fetal capillaries, fetal connective tissue, maternal epithelium or maternal connective tissue in the placentome (Table 3). Volumes of trophoblasts, maternal epithelium and maternal connective tissues in placenta were lower (*P *<* *0.05) in the Hx compared with the Nx groups, and the volumes of fetal connective tissue and maternal connective tissue in placenta were lower in the Cx compared with the Control groups (*P *<* *0.05; Table 3). There was no effect of Cx or Hx on volumes of fetal capillaries and maternal capillaries in placenta (Table 3).

**Table 3:** Effects of carunclectomy and hypoxemia on the volume density (*V*_d_) and volumes of fetal and maternal components in the placentome and placenta during late gestation

	Control		Cx	
	Nx(*n* = 5)	Hx(*n* = 5)	Nx(*n* = 3)	Hx(*n* = 3)
Trophoblast				
*V*_d_	0.440 ± 0.033	0.494 ± 0.048	0.510 ± 0.035	0.450 ± 0.043
Volume in placentome (cm^3^)	3.49 ± 0.42	2.94 ± 0.56	6.00 ± 0.80^b^	4.71 ± 1.35^b^
Volume in placenta (cm^3^)	214.9 ± 27.7	156.3 ± 23.2^a^	170.3 ± 38.0	97.8 ± 11.2^a^
Fetal capillaries				
*V*_d_	0.013 ± 0.003	0.027 ± 0.006	0.021 ± 0.002	0.018 ± 0.003
Volume in placentome (cm^3^)	0.111 ± 0.035	0.219 ± 0.082	0.247 ± 0.018	0.174 ± 0.041
Volume in placenta (cm^3^)	6.07 ± 1.60	9.21 ± 3.09	7.28 ± 2.13	3.84 ± 0.81
Fetal connective tissue				
*V*_d_	0.073 ± 0.016	0.124 ± 0.022	0.069 ± 0.011	0.064 ± 0.008
Volume in placentome (cm^3^)	0.625 ± 0.181	0.801 ± 0.094	0.784 ± 0.083	0.769 ± 0.337
Volume in placenta (cm^3^)	35.4 ± 8.9	35.8 ± 2.4	22.8 ± 6.8^b^	13.9 ± 2.1^b^
Maternal epithelium				
*V*_d_	0.249 ± 0.033	0.192 ± 0.035	0.241 ± 0.021	0.267 ± 0.014
Volume in placentome (cm^3^)	2.01 ± 0.35	1.49 ± 0.51	2.78 ± 0.13	3.07 ± 1.30
Volume in placenta (cm^3^)	117.0 ± 13.3	66.3 ± 16.4^a^	78.2 ± 15.2	57.7 ± 2.3^a^
Maternal capillaries				
*V*_d_	0.018 ± 0.005	0.013 ± 0.003	0.019 ± 0.003	0.023 ± 0.009
Volume in placentome (cm^3^)	0.148 ± 0.044	0.085 ± 0.020	0.225 ± 0.027^b^	0.191 ± 0.015^b^
Volume in placenta (cm^3^)	8.27 ± 1.67	4.41 ± 1.38	6.10 ± 0.72	4.94 ± 1.88
Maternal connective tissue				
*V*_d_	0.206 ± 0.009	0.150 ± 0.027	0.140 ± 0.003	0.179 ± 0.036
Volume in placentome (cm^3^)	1.66 ± 0.20	1.11 ± 0.34	1.63 ± 0.06	2.28 ± 1.27
Volume in placenta (cm^3^)	99.4 ± 10.1	48.5 ± 10.7^a^	46.4 ± 10.3^b^	38.6 ± 7.4^a^^,^ b

Values are mean ± SEM. Cx, carunclectomy; Nx, normoxemic group; Hx, hypoxemic group

a Denotes an effect of hypoxemia (Nx vs. Hx)

b Denotes an effect of Cx treatment (Control vs. Cx); *P *<* *0.05.

There was no difference in surface density and barrier thickness between the Control and Cx groups independent of whether the fetus was Nx or Hx (Table 4). The surface area for exchange in the placentome was higher (*P *<* *0.05) in the Cx compared with the Control groups regardless of whether the fetus was Nx or Hx (Table 4). The surface area for exchange in the placenta was lower (*P *<* *0.05) in the Hx compared with the Nx groups in both Control and Cx fetuses (Table 4).

**Table 4:** Effects of carunclectomy and hypoxemia on trophoblast exchange surface during late gestation

	Control		Cx	
	Nx(*n* = 5)	Hx(*n* = 5)	Nx(*n* = 3)	Hx(*n* = 3)
Surface density (cm^2^ g^−1^)	271 ± 31	262 ± 12	298 ± 18	260 ± 22
Surface area of placentome (m^2^)	0.214 ± 0.032	0.147 ± 0.020	0.350 ± 0.043^b^	0.273 ± 0.081^b^
Surface area of placenta (m^2^)	13.23 ± 1.99	8.55 ± 1.46^a^	9.74 ± 1.74	5.64 ± 0.49^a^
Barrier thickness (μm)	16.60 ± 0.90	19.17 ± 2.54	17.15 ± 0.97	17.28 ± 0.68

Values are mean ± SEM. Cx, carunclectomy; Nx, normoxemic group; Hx, hypoxemic group

a Denotes an effect of hypoxemia (Nx vs. Hx)

b Denotes an effect of Cx treatment (Control vs. Cx); *P *<* *0.05.

### Effects of carunclectomy and fetal hypoxemia on mRNA expression in the placenta

There was no effect of fetal sex or any interaction between fetal sex and Cx or Hx on the mRNA expression of any of the genes.

#### IGFs and receptors

Placental *IGF2* mRNA expression was higher in the Cx groups compared with the Control groups independent of whether the fetus was Nx or Hx (*P *<* *0.05; Table 5). There was no difference in placental *IGF1*,* IGF1R*, and *IGF2R* mRNA expression between the four groups (Table 5).

**Table 5:** The effects of carunclectomy and hypoxemia on the mRNA expression of IGFs and their receptors, HIFs, regulators of apoptosis, and autophagy in late gestational sheep fetuses

	Control		Cx	
	Nx(*n* = 20)	Hx(*n* = 8)	Nx(*n* = 12)	Hx(*n* = 16)
IGFs & their receptors				
* IGF1*	0.007 ± 0.001	0.007 ± 0.001	0.005 ± 0.001	0.006 ± 0.001
* IGF2*	0.963 ± 0.077	0.998 ± 0.175	1.613 ± 0.160^b^	1.202 ± 0.153^b^
* IGF1R*	0.058 ± 0.007	0.048 ± 0.005	0.051 ± 0.009	0.054 ± 0.006
* IGF2R*	0.055 ± 0.005	0.062 ± 0.004	0.055 ± 0.007	0.063 ± 0.005
HIFs				
* HIF1A*	0.255 ± 0.016	0.173 ± 0.009^a^	0.212 ± 0.018	0.248 ± 0.024^b^
* HIF1B*	0.096 ± 0.005	0.081 ± 0.005	0.108 ± 0.009	0.100 ± 0.009
* HIF2A*	0.339 ± 0.031	0.227 ± 0.032	0.335 ± 0.043	0.354 ± 0.047
* HIF3A*	0.005 ± 0.001	0.008 ± 0.001^a^	0.006 ± 0.001	0.007 ± 0.001^a^
Apoptosis				
* BAX*	0.059 ± 0.004	0.056 ± 0.005	0.053 ± 0.003	0.056 ± 0.003
* BCL2*	0.0026 ± 0.0003	0.0023 ± 0.0003	0.0029 ± 0.0006	0.0024 ± 0.0003
* p53*	0.031 ± 0.001	0.028 ± 0.003	0.026 ± 0.001^b^	0.030 ± 0.001
Autophagy				
* BECLIN1*	0.076 ± 0.004	0.066 ± 0.005	0.073 ± 0.003	0.074 ± 0.007
* LC3B*	0.102 ± 0.003	0.081 ± 0.007^a^	0.110 ± 0.004^b^	0.098 ± 0.004^a^^,^ b
* LAMP1*	0.116 ± 0.008	0.157 ± 0.021^a^	0.123 ± 0.009	0.117 ± 0.007^b^

Cx, carunclectomy. Nx, normoxemic group. Hx, hypoxemic group. Values are mean ± SEM.

a Denotes an effect of hypoxemia (Nx vs. Hx);

b denotes an effect of Cx treatment (Control vs. Cx). *P *<* *0.05.

#### Hypoxia‐inducible factors

There was an interaction between the effects of Cx and Hx on placental *HIF1A* mRNA expression (*P *<* *0.05; Table 5). *HIF1A* mRNA expression was lower in the Control+Hx group compared with the Control+Nx group, but was higher in the Cx+Hx group compared with the Control+Hx group. In contrast, there was no difference in placental mRNA expression of *HIF1B* and *HIF2A* between the four groups (Table 5). Placental mRNA expression of *HIF3A* was higher in the Hx compared with the Nx groups in both Control and Cx fetuses (*P *<* *0.05; Table 5).

#### Genes involved in vasculogenesis and angiogenesis

**Figure 4:**
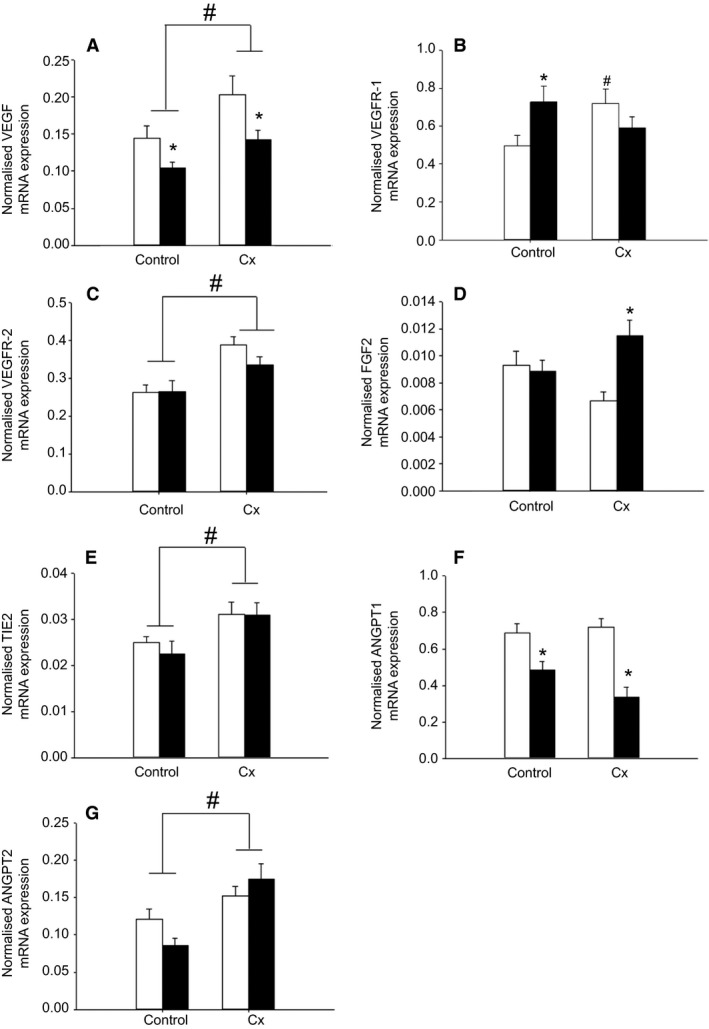
The effects of carunclectomy and hypoxemia on placental mRNA expression of *VEGF* (A), *VEGFR‐1* (B), *VEGFR‐2* (C), *FGF2* (D), *TIE2* (E), *ANGPT1,* (F) and *ANGPT2* (G). Nx, Normoxemic group, open bars; Hx, Hypoxemic group, closed bars; *, denotes an effect of hypoxemia (Nx vs. Hx); ^#^, denotes an effect of Cx treatment (Control vs. Cx). *P *<* *0.05. VEGF, vascular endothelial growth factor.

Placental *VEGF* mRNA expression was higher (*P *<* *0.05) in the Cx compared with the Control groups (*P *<* *0.05), but was lower (*P *<* *0.05) in the Hx compared with the Nx groups (Fig. 4). Placental *VEGFR‐2*,* TIE2*, and *ANGPT2* mRNA expression was higher in the Cx compared with the Control groups independent of whether the fetus was Nx or Hx (*P *<* *0.05; Fig. 4). Placental *ANGPT1* mRNA expression was lower in the Hx compared with the Nx groups in both Control and Cx fetuses (*P *<* *0.05; Fig. 4). There was an interaction between the effects of Cx and Hx on placental *VEGFR‐1* and *FGF2* mRNA expression (*P *<* *0.05, Fig. 4). Placental *VEGFR‐1* mRNA was higher in the Control+Hx and Cx+Nx groups compared with the Control+Nx group. Placental *FGF2* mRNA expression was higher in the Cx+Hx compared with the Cx+Nx group.

**Figure 5:**
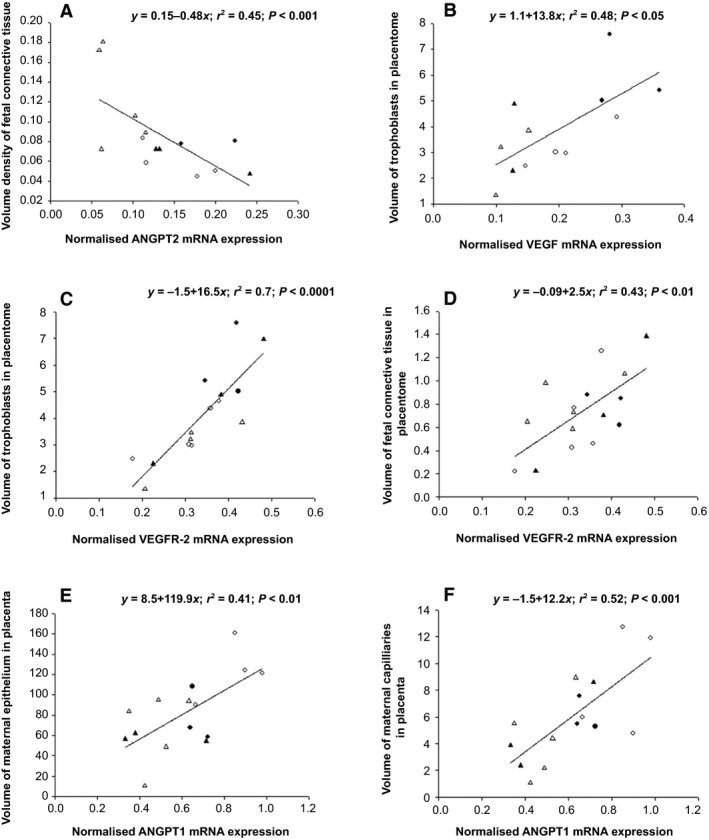
The relationships between placental *ANGPT2* mRNA expression and volume density of fetal connective tissue (A), between *VEGF* mRNA expression and volume of trophoblasts in the placentome (B), between *VEGFR‐2* mRNA expression and volume of trophoblasts in the placentome (C), between *VEGFR‐2* mRNA expression and volume of fetal connective tissue in the placentome (D), between *ANGPT1* mRNA expression and volume of maternal epithelium in the placenta (E), and between *ANGPT1* mRNA expression and volume of maternal capillaries in the placenta (F) in the Control+Nx (○), Control+Hx (Δ), Cx+Nx (●) and Cx+Hx (▲) groups. VEGF, vascular endothelial growth factor.

When all treatment groups were considered, there were significant relationships between placental *ANGPT2* mRNA expression and volume density of fetal connective tissue, between *VEGF* mRNA expression and volume of trophoblasts in the placentome, between *VEGFR‐2* mRNA expression and volumes of trophoblasts and fetal connective tissue in the placentome, and between *ANGPT1* mRNA expression and volumes of maternal epithelium and maternal capillaries in the placenta (Fig. 5).

#### Glucose, amino acid and fatty acid transporters

**Figure 6:**
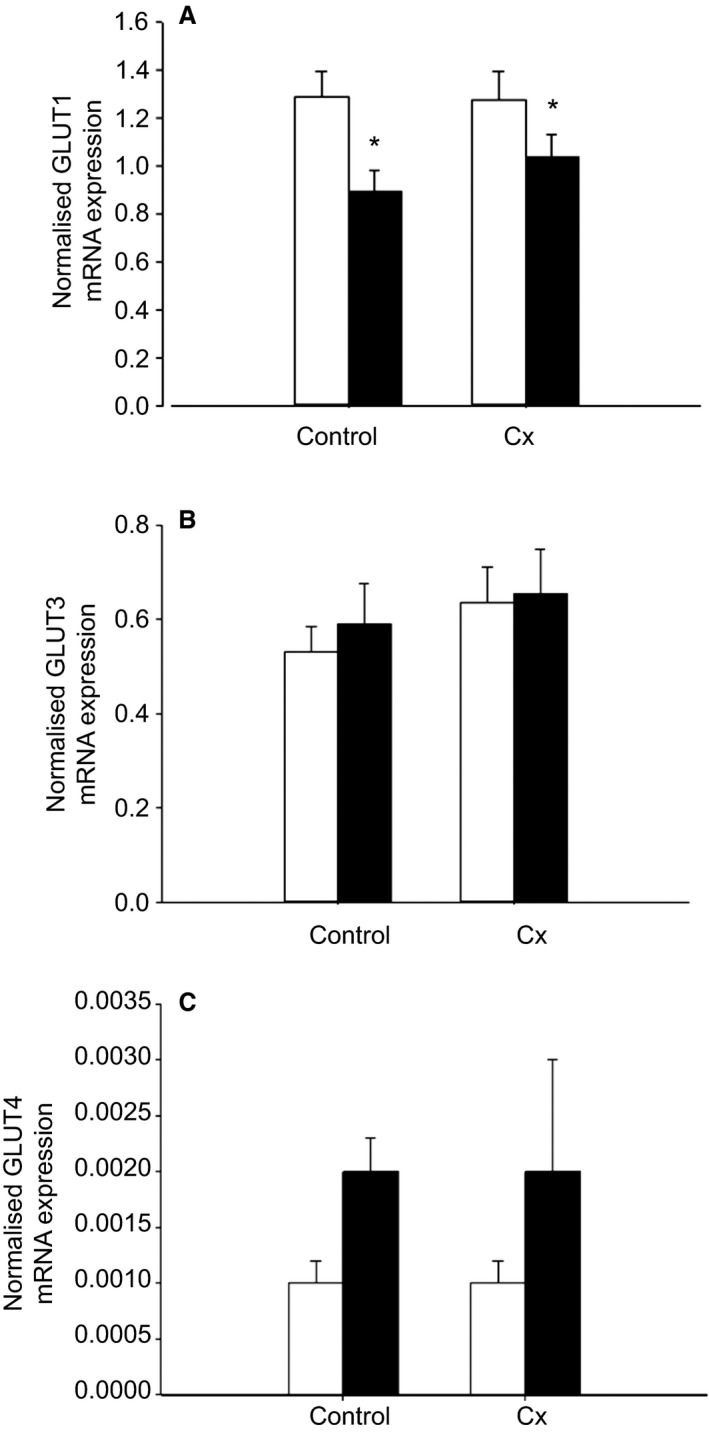
The effects of carunclectomy and hypoxemia on placental mRNA expression of *GLUT1* (A), *GLUT3* (B) and *GLUT4* (C). Nx, Normoxemic group, open bars; Hx, Hypoxemic group, closed bars; *, denotes an effect of hypoxemia (Nx vs Hx). *P *<* *0.05.

**Figure 7:**
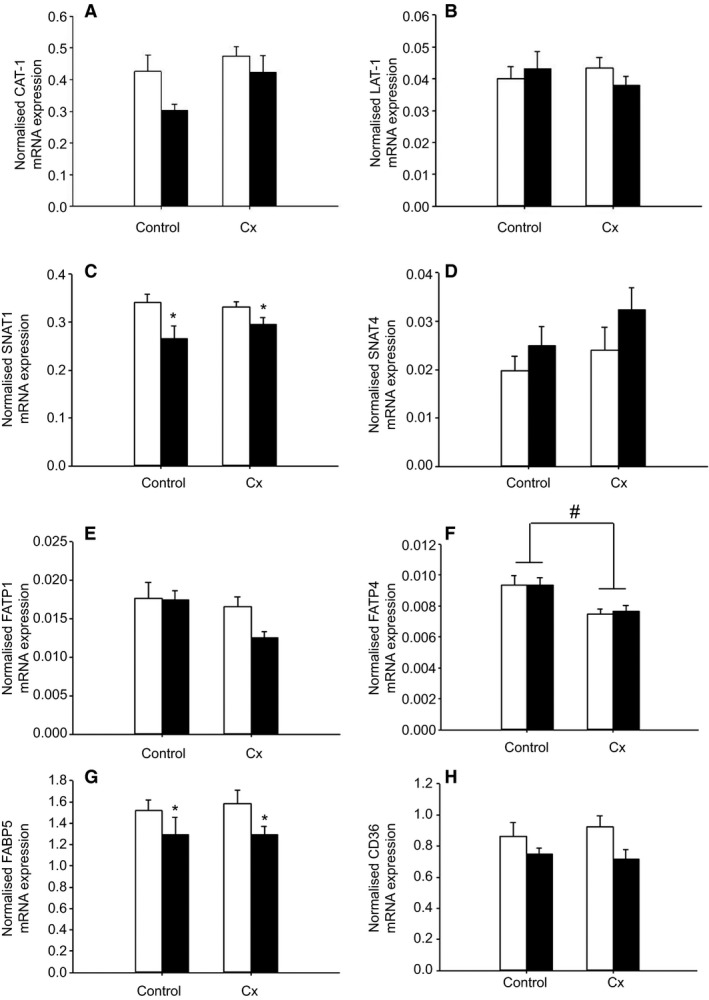
The effects of carunclectomy and hypoxemia on placental mRNA expression of *CAT‐1* (A), *LAT‐1* (B), *SNAT1* (C), *SNAT4* (D), *FATP1* (E), *FATP4* (F), *FABP5* (G) and *CD36* (H). Nx, Normoxemic group, open bars; Hx, Hypoxemic group, closed bars; *, denotes an effect of hypoxemia (Nx vs Hx); #, denotes an effect of Cx treatment (Control vs Cx). *P *<* *0.05.

Placental *GLUT1* mRNA expression was lower (*P *<* *0.05) in the Hx compared with the Nx groups, regardless of whether or not they were Control or Cx (Fig. 6). There was no difference in placental *GLUT3* or *GLUT4* mRNA expression between the four groups (Fig. 6). Placental *FATP4* mRNA expression was lower (*P *<* *0.05) in the Cx compared with Control groups independent of whether the fetus was Nx or Hx (Fig. 7). Placental mRNA expression of *SNAT1* and *FABP5* was lower (*P *<* *0.05) in the Hx compared with the Nx groups in both Control and Cx fetuses (Fig. 7). There was no difference in placental mRNA expression of *CAT‐1*,* LAT‐1*,* SNAT4*,* FATP1*, and *CD36* between the four groups (Fig. 7).

#### Potential glucocorticoid exposure

**Figure 8:**
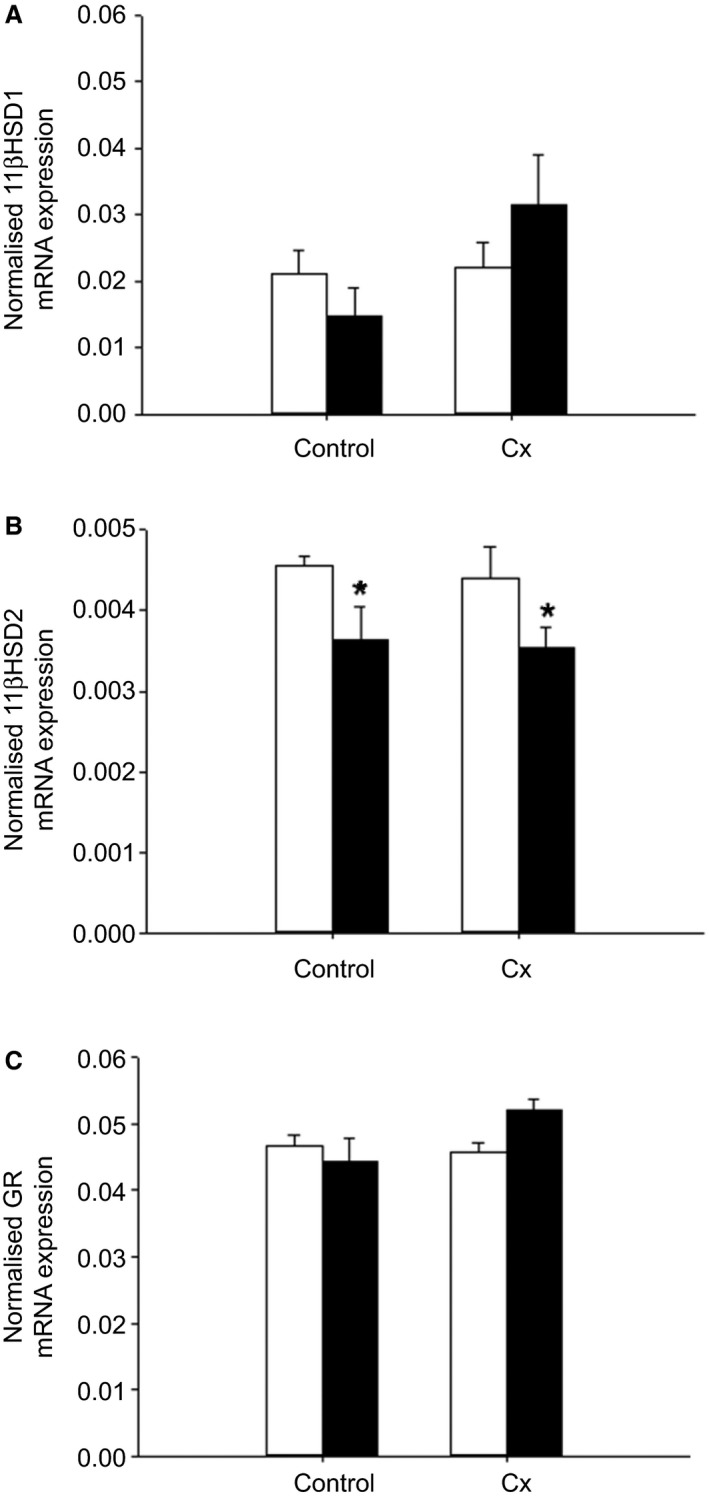
The effects of carunclectomy and hypoxemia on placental mRNA expression of *11βHSD1* (A), *11βHSD2* (B) and *GR* (C). Nx, Normoxemic group, open bars; Hx, Hypoxemic group, closed bars; *, denotes an effect of hypoxemia (Nx vs. Hx). *P *<* *0.05.

Placental *11βHSD2* mRNA expression was lower in the Hx compared with the Nx groups in both Control and Cx fetuses (*P *<* *0.05; Fig. 8). There was no difference in placental mRNA expression of *11βHSD1* and *GR* between the four groups (Fig. 8).

#### Apoptosis and autophagy

There was no difference in placental mRNA expression of regulators of apoptosis, including *BAX* and *BCL2*, as well as autophagy including *BECLIN1*, between the 4 groups (Table 5). Placental *p53* mRNA was lower (*P *<* *0.05) in the Cx+Nx group compared with the Control+Nx group (Table 5). Placental *LC3B* mRNA expression was higher (*P *<* *0.05) in the Cx compared with the Control groups, but lower (*P *<* *0.05) in the Hx compared with the Nx groups (Table 5). There was an interaction between the effects of Cx and Hx on placental *LAMP1* mRNA expression (*P *<* *0.05; Table 5), specifically, mRNA expression was higher in the Control+Hx group compared with the Control+Nx group, but was lower in the Cx+Hx group compared with the Control+Hx group.

## Discussion

**Figure 9:**
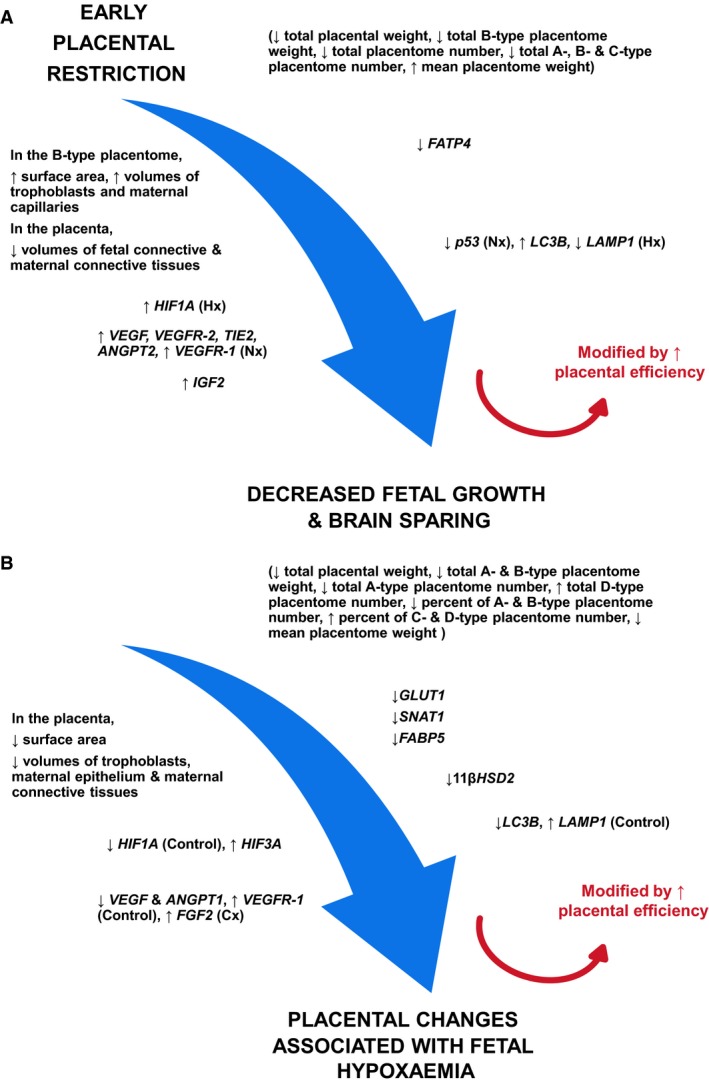
Summary of morphological and molecular changes in the placenta of Cx (A) and Hx (B) groups. ↑, increase; ↓, decrease. Nx, Hx, Control and Cx denote the changes in these specific groups only.

This study has shown that early restriction of placental growth affects morphological and functional characteristics of the placenta in late gestation, independently of whether or not the fetus becomes hypoxemic (Fig. 9). In addition, independently of whether experimental restriction of placental growth occurred, there is a distinct set of morphological and gene expression changes in the placenta that are only present in fetuses that were hypoxemic in late gestation (Fig. 9).

### Effects of carunclectomy and hypoxemia on placental weight and morphology

Both Cx and Hx resulted in a decrease in total placental weight and the number of specific types of placentomes while Hx was also related to an increase in the number of D type placentomes. These results indicate a shift from inverted to everted placentome types in late gestation. Placental efficiency was increased in both Cx and Hx groups, suggesting placental adaptation in response to suboptimal placental function, in both early restriction of placental growth and the emergence of placental insufficiency. These findings were consistent with previous studies in sheep models of placental insufficiency, in which uterine carunclectomy and hyperthermia‐induced placental restriction resulted in reduced placental weight and IUGR (McMillen et al. 2001; Limesand et al. 2006; Regnault et al. 2007; Poudel et al. 2015) as well as increased placental efficiency (Regnault et al. 2003; Fowden et al. 2006; Poudel et al. 2015). Our study has reinforced the previous proposal that placentome eversion is an adaptation to placental restriction, which occurs to increase the efficiency of placental substrate transfer to the fetus (Owens et al. 1987a,b; Robinson et al. 1997). Interestingly, it has been reported that early exposure to high plasma cortisol concentrations decreases the proportion of type C and D placentomes, which indicates that developmental shifts in placentome classification are not restricted to eversion (Ward et al. 2006). However, in our sheep model of carunclectomy, we have shown previously that there were no separate effects of either carunclectomy or hypoxemia on fetal plasma cortisol concentrations, suggesting that the developmental shifts in placentome types may depend on the duration and degree of circulating or intraplacental glucocorticoid exposure (Butler et al. 2002; Poudel et al. 2015).

Early restriction of placental growth resulted in an increase in surface area and volumes of trophoblasts and maternal capillaries in the inverted placentome, which may improve maternal and fetal exchange and an adaptation to the reduced total placentome number due to carunclectomy. This is consistent with previous studies in this model that found an increase in the volume density of the trophoblast and the feto‐maternal syncytium within the placentomes (Robinson et al. 1995). When all types of placentomes are considered, Cx resulted in a decrease in the placental volumes of fetal and maternal connective tissues, whereas Hx was related to a decrease in placental surface area and volumes of trophoblasts, maternal epithelium and connective tissues. These data are in agreement with previous reports in humans that have shown a decrease in surface area, volume, and number of terminal villi and capillaries in placentas from IUGR compared with normal pregnancies (Krebs et al. 1996; Challis et al. 2000; Mayhew et al. 2003). Placentomes undergo progressive structural remodeling, and all cell layers attenuate in both fetal and maternal tissues resulting in fetal and maternal capillaries in close proximity to allow increased capacity for substrate transfer in the placenta when fetal demand for nutrients is high in mid‐late gestation (Ward et al. 2006). Therefore, the attenuation of the cell layers in the placenta of the Cx and Hx groups may represent accelerated placental maturation to facilitate improved placental transport.

This accelerated placental maturation may be caused by the increase in placental *IGF2* mRNA expression due to Cx, but not Hx. This increase in placental *IGF2* expression may be an adaptive response to early restriction of placental growth and may subsequently play a role in promoting vasculogenesis and angiogenesis in the placenta. Similarly, placental *IGF2* and *IGF1* mRNA expression is increased at both 55 and 90 days of gestation in the hyperthermia sheep model of IUGR (de Vrijer et al. 2006). Furthermore, in guinea pigs, exogenous treatment of the mother with IGF2 in early gestation increased the volume of placental labyrinth, trophoblast and maternal blood space within the labyrinth and total surface area of trophoblast for exchange, which further suggests that IGF2 may enhance morphological and functional development of the placenta and nutrient delivery (Sferruzzi‐Perri et al. 2008).

### Effects of carunclectomy and hypoxemia on placental expression of genes involved in vasculogenesis and angiogenesis

Placental mRNA expression of *HIF1A, VEGF*,* VEGFR‐1*,* VEGFR‐2*,* ANGPT2*, and *TIE2* was significantly increased in the Cx groups compared with the Controls, which may lead to vessel instability, angiogenesis and vessel remodeling. There were also significant relationships between placental angiogenic growth factor mRNA expression and volumes of specific fetal and maternal components in the placentome and placenta. These data suggest that the accelerated placental maturation upon early restriction of placental growth and late emergence of placental insufficiency may be through the regulation of placental angiogenic growth factors. Similarly, VEGF‐A and bFGF immunostaining was significantly higher in cytotrophoblasts, syncytiotrophoblasts, extravillous trophoblasts, vascular smooth muscle cells, chorionic villous stromal cells, and villous vascular endothelial cells of human IUGR placentas compared with those collected from normal term pregnancies (Barut et al. 2010). In addition, hyperthermia‐induced IUGR resulted in increased uterine blood flow and increased *VEGF*,* ANGPT1*,* ANGPT2*, and *TIE‐2* mRNA expression in the placentome in early gestation in sheep (Regnault et al. 2002, 2003; Hagen et al. 2005). On the other hand, there were additional complex placental changes related to Hx, including a decrease in placental mRNA expression of *HIF1A*,* VEGF*, and *ANGPT1*, as well as an increase in *HIF3A, VEGFR‐1*, and *FGF2* mRNA. The reduced mRNA expression of *HIF1A* and genes with hypoxia response elements, *VEGF* and *ANGPT1*, may represent a partial compensatory response to chronic hypoxemia. This is supported by the finding that 3 day exposure to low oxygen decreased *HIF1A*, but not *HIF2A* mRNA expression in cultured murine ectoplacental cones (Pringle et al. 2007).

### Effects of carunclectomy and hypoxemia on placental mRNA expression of glucose, amino acid and fatty acid transporters

In this study, Hx was associated with a decrease in placental *GLUT1* mRNA expression in late gestation, while there was no effect of Cx on placental glucose transporters. Decreased placental GLUT1 gene and protein expression has also been found in other studies. Chronic hypoxemia resulted in a decrease in GLUT1 protein abundance in the basal membrane, but not microvillous plasma membrane of the placenta, subsequently leading to reduced nutrient supply and fetal weight in high‐altitude pregnancies (Zamudio et al. 2006). In mice, maternal chronic hypoxia resulted in a decrease in placental GLUT1 mRNA and protein expression in female fetuses, but no change in placental *GLUT3* mRNA expression in both female and male fetuses during mid‐ to late gestation (Cuffe et al. 2014). Therefore, our data suggested that hypoxemia may result in impaired glucose transfer.

Cx resulted in a decrease in placental *FATP4* mRNA expression, whereas Hx was related to a decrease in placental *SNAT1* and *FABP5* mRNA expression in late gestation. Alterations of placental amino acid and fatty acid transporters occur in IUGR pregnancies and/or under hypoxic conditions (Nelson et al. 2003; Regnault et al. 2005; Biron‐Shental et al. 2007; Cuffe et al. 2014). Maternal hyperthermia resulted in an increase in placental *LAT‐1* and *LAT‐2* mRNA expression in IUGR sheep fetuses where placental and fetal weight was reduced by 25% compared with Controls (Regnault et al. 2005). Placental system A transport and activity was reduced by hypoxia in full‐term human trophoblasts (Nelson et al. 2003). In mice, maternal hypoxia resulted in a decrease in placental *SNAT* mRNA expression in both female and male fetuses during mid to late gestation (Cuffe et al. 2014). Interestingly, hypoxia elevated the expression of FABP1, ‐3, and ‐4, but not FABP5 in term human trophoblasts, suggesting that FABPs may play a role in fat accumulation and metabolism in the hypoxic placenta (Biron‐Shental et al. 2007). Collectively, it has been suggested that the regulation of placental nutrient transport capacity in IUGR pregnancies and/or under hypoxic conditions may be species‐specific and different between in vivo and in vitro studies, and depend on the timing and type of insult.

### Effects of carunclectomy and hypoxemia on potential glucocorticoid exposure

Placental *11βHSD2* mRNA expression was decreased in the Hx compared to Nx groups, although there was no effect of Cx alone on placental *GR*,* 11βHSD1* and *11βHSD2* mRNA expression. This suggests that increased intra‐placental glucocorticoid concentrations may play a role in regulating the placental insufficiency in late gestation responsible for chronic substrate restriction and subsequent fetal hypoxemia. This is consistent with a previous report that maternal hypoxia during mid‐ to late gestation in mice decreased placental 11*β*HSD2 mRNA and protein expression (Cuffe et al. 2014). Similarly in human pregnancies, placental 11*β*HSD2 expression and activity were significantly reduced in deliveries complicated by IUGR compared with the term deliveries and with appropriately grown preterm deliveries, although it was not reported whether or not these IUGR fetuses were hypoxemic (Shams et al. 1998).

### Effects of carunclectomy and hypoxemia on genes regulating apoptosis and autophagy

There was no difference in placental mRNA expression of apoptotic and anti‐apoptotic markers between the four groups, although placental *p53* mRNA was lower in the Cx group compared with the Control group in the Nx fetuses. These data were inconsistent with the previous studies that placentas from women with IUGR pregnancies showed enhanced apoptosis compared to Controls (Smith et al. 1997; Erel et al. 2001). Cultured trophoblasts exposed to hypoxia (1–2%) alone showed upregulation of apoptotic p53 activity and BAX expression and decreased expression of anti‐apoptotic BCL‐2 (Levy et al. 2000). In addition, genes involved in regulating autophagy were altered by Cx and Hx. In this study, Cx increased placental *LC3B* and decreased *LAMP1* mRNA expression, whereas Hx decreased *LC3B* and increased *LAMP1* mRNA expression during late gestation. In contrast, women delivering an IUGR baby had increased placental levels of autophagy‐related proteins, including LC3B, beclin‐1 and p53 compared with pregnant women delivering a normally grown baby (Hung et al. 2012). Such a discrepancy in placental apoptosis and autophagy may be due to the cause, timing and degree of hypoxemia (Morrison 2008).

One limitation of this study is that all analyses were performed in the inverted placentomes. However, previous studies have shown that there is no effect of placentome type on the expression of a large range of vasculogenic and angiogenic genes and that size may be related to function (Vonnahme et al. 2008). Furthermore, although cortisol decreases the proportion of type D placentomes (Ward et al. 2006), it does not change the umbilical uptake of lactate or oxygen relative to fetal weight between placentome types. In fact, there is no difference in umbilical uptake of glucose, oxygen and lactate relative to fetal weight bethween placentome types in the saline‐infused controls (Ward et al. 2006). A second limitation of the study is that the cotyledonary structure of the sheep placenta differs from the discoid human placenta. However, the sheep has a structurally similar villous type of the placenta to that in the human, which is reflected by the architecture of the fetal villous tree and terminal vessels (Beckett et al. 2014). In addition, our sheep IUGR model induced by carunclectomy has been shown to be a good animal model system to study human IUGR (Alexander 1964; McMillen et al. 2001; Morrison 2008). The molecular mechanisms identified in this study might thus improve our understanding and facilitate development of therapeutic interventions.

## Perspectives and significance

Experimental restriction of placental growth results in IUGR fetuses that are chronically hypoxemic and hypoglycemic. This study demonstrates that the IUGR fetus displays an accelerated placental maturation of capillarization and angiogenesis, as well as evidence of poor nutrient transport capacity from the maternal to the fetal circulation and failure to maintain placental and fetal growth. Early restriction of placental growth induced by carunclectomy resulted in decreased volumes of fetal and maternal connective tissues in the placenta and in increased placental mRNA expression of *IGF2* and angiogenic factors, which subsequently led to decreased fetal weight and brainsparing. These morphological and functional characteristics of the placenta in late gestation are present independently of whether the fetus becomes hypoxemic. We also identified a distinct set of placental morphological and gene expression changes, including reduced volumes of trophoblast, maternal epithelium and maternal connective tissues in the placenta, a decrease in placental *GLUT1* and *11βHSD2* mRNA in the fetuses that were hypoxemic in late gestation, independent of whether or not surgical carunclectomy had been performed. Therefore, this study provides further understanding of the different placental cellular and molecular mechanisms that are present in early placental restriction and in the emergence of later placental insufficiency and this may facilitate the development of therapeutic interventions.

## Conflict of Interest

The authors have no conflict of interest.

## References

[phy213049-bib-0001] Alexander, G. 1964 Studies on the placenta of the sheep (ovis aries L.). Effect of surgical reduction in the number of caruncles. J. Reprod. Fertil. 30:307–322. 10.1530/jrf.0.0070307 14180724

[phy213049-bib-0002] Bamfo, J. E. , and A. O. Odibo . 2011 Diagnosis and management of fetal growth restriction. J. Pregnancy 2011:640715. 21547092 10.1155/2011/640715 PMC3087156

[phy213049-bib-0003] Barker, D. J. , P. D. Gluckman , K. M. Godfrey , J. E. Harding , J. A. Owens , and J. S. Robinson . 1993 Fetal nutrition and cardiovascular disease in adult life. Lancet 341:938–941. 8096277 10.1016/0140-6736(93)91224-a

[phy213049-bib-0004] Barker, D. J. , J. Gelow , K. Thornburg , C. Osmond , E. Kajantie , and J. G. Eriksson . 2010 The early origins of chronic heart failure: impaired placental growth and initiation of insulin resistance in childhood. Eur. J. Heart Fail. 12:819–825. 20504866 10.1093/eurjhf/hfq069 PMC5477852

[phy213049-bib-0005] Barut, F. , A. Barut , B. D. Gun , N. O. Kandemir , M. I. Harma , M. Harma , et al. 2010 Intrauterine growth restriction and placental angiogenesis. Diagn. Pathol. 5:24. 20412591 10.1186/1746-1596-5-24 PMC2865442

[phy213049-bib-0006] Beckett, E. M. , O. Astapova , T. L. Steckler , A. Veiga‐Lopez , and V. Padmanabhan . 2014 Developmental programing: impact of testosterone on placental differentiation. Reproduction 148:199–209. 24840528 10.1530/REP-14-0055 PMC4091887

[phy213049-bib-0007] Biron‐Shental, T. , W. T. Schaiff , C. K. Ratajczak , I. Bildirici , D. M. Nelson , and Y. Sadovsky . 2007 Hypoxia regulates the expression of fatty acid‐binding proteins in primary term human trophoblasts. Am. J. Obstet. Gynecol. 197: 516.e511–516.e516. 17826730 10.1016/j.ajog.2007.03.066 PMC2151846

[phy213049-bib-0008] Botting, K. J. , I. C. McMillen , H. Forbes , J. R. Nyengaard , and J. L. Morrison . 2014 Chronic hypoxemia in late gestation decreases cardiomyocyte number but does not change expression of hypoxia‐responsive genes. J. Am. Heart Assoc. 3. 10.1161/JAHA.113.000531 PMC4310356 25085511

[phy213049-bib-0009] Bustin, S. A. , V. Benes , J. A. Garson , J. Hellemans , J. Huggett , M. Kubista , et al. 2009 The MIQE guidelines: minimum information for publication of quantitative real‐time PCR experiments. Clin. Chem. 55:611–622. 19246619 10.1373/clinchem.2008.112797

[phy213049-bib-0010] Butler, T. G. , J. Schwartz , and I. C. McMillen . 2002 Differential effects of the early and late intrauterine environment on corticotrophic cell development. J. Clin. Invest. 110:783–791. 12235109 10.1172/JCI15563 PMC151129

[phy213049-bib-0011] Challis, D. E. , C. D. Pfarrer , J. W. Ritchie , G. Koren , and S. L. Adamson . 2000 Glucose metabolism is elevated and vascular resistance and maternofetal transfer is normal in perfused placental cotyledons from severely growth‐restricted fetuses. Pediatr. Res. 47:309–315. 10709728 10.1203/00006450-200003000-00005

[phy213049-bib-0012] Cuffe, J. S. , S. L. Walton , R. R. Singh , J. G. Spiers , H. Bielefeldt‐Ohmann , L. Wilkinson , et al. 2014 Mid‐ to late term hypoxia in the mouse alters placental morphology, glucocorticoid regulatory pathways and nutrient transporters in a sex‐specific manner. J. Physiol. 592:3127–3141. 24801305 10.1113/jphysiol.2014.272856 PMC4214664

[phy213049-bib-0013] Danielson, L. , I. C. IMcMillen , J. L. Dyer , J. L. 2Morrison . 2005 Restriction of placental growth results in greater hypotensive response to *α*‐adrenergic blockade in sheep during late gestation. J. Physiol. 563:611–620. 15649982 10.1113/jphysiol.2004.080523 PMC1665578

[phy213049-bib-0014] Dubova, E. A. , K. A. Pavlov , G. V. Kulikova , A. I. Shchegolev , and G. T. Sukhikh . 2013 Glucose transporters expression in the placental terminal villi of preeclampsia and intrauterine growth retardation complicated pregnancies. Health 5:100–104.

[phy213049-bib-0015] Dyer, J. L. , I. C. McMillen , K. E. Warnes , and J. L. Morrison . 2009 No evidence for an enhanced role of endothelial nitric oxide in the maintenance of arterial blood pressure in the IUGR sheep fetus. Placenta 30:705–710. 19515421 10.1016/j.placenta.2009.05.003

[phy213049-bib-0016] Edwards, L. J. , G. Simonetta , J. A. Owens , J. S. Robinson , and I. C. McMillen . 1999 Restriction of placental and fetal growth in sheep alters fetal blood pressure responses to angiotension II and captopril. J. Physiol. 515:897–904. 10066914 10.1111/j.1469-7793.1999.897ab.x PMC2269199

[phy213049-bib-0017] Erel, C. T. , B. Dane , Z. Calay , S. Kaleli , and K. Aydinli . 2001 Apoptosis in the placenta of pregnancies complicated with IUGR. Int. J. Gynaecol. Obstet. 73:229–235. 11376669 10.1016/s0020-7292(01)00373-3

[phy213049-bib-0018] Fletcher, C. J. , C. T. Roberts , K. M. Hartwich , S. K. Walker , and I. C. McMillen . 2007 Somatic cell nuclear transfer in the sheep induces placental defects that likely precede fetal demise. Reproduction 133:243–255. 17244750 10.1530/rep.1.01203

[phy213049-bib-0019] Fowden, A. L. , J. W. Ward , F. P. B. Wooding , A. J. Forhead , and M. Constancia . 2006 Programming placental nutrient transfer capacity. J. Physiol. 572:5–15. 16439433 10.1113/jphysiol.2005.104141 PMC1779642

[phy213049-bib-0020] Hagen, A. S. , R. J. Orbus , R. B. Wilkening , T. R. Regnault , and R. V. Anthony . 2005 Placental expression of angiopoietin‐1, angiopoietin‐2 and tie‐2 during placental development in an ovine model of placental insufficiency‐fetal growth restriction. Pediatr. Res. 58:1228–1232. 16306198 10.1203/01.pdr.0000185266.23265.87

[phy213049-bib-0021] Hung, T. H. , S. F. Chen , L. M. Lo , M. J. Li , Y. L. Yeh , and T. T. Hsieh . 2012 Increased autophagy in placentas of intrauterine growth‐restricted pregnancies. PLoS ONE 7:e40957. 22815878 10.1371/journal.pone.0040957 PMC3397998

[phy213049-bib-0022] Janzen, C. , M. Y. Lei , J. Cho , P. Sullivan , B. C. Shin , and S. U. Devaskar . 2013 Placental glucose transporter 3 (GLUT3) is up‐regulated in human pregnancies complicated by late‐onset intrauterine growth restriction. Placenta 34:1072–1078. 24011442 10.1016/j.placenta.2013.08.010 PMC3843645

[phy213049-bib-0023] Krebs, C. , L. M. Macara , R. Leiser , A. W. Bowman , I. A. Greer , and J. C. Kingdom . 1996 Intrauterine growth restriction with absent end‐diastolic flow velocity in the umbilical artery is associated with maldevelopment of the placental terminal villous tree. Am. J. Obstet. Gynecol. 175:1534–1542. 8987938 10.1016/s0002-9378(96)70103-5

[phy213049-bib-0024] Levy, R. , S. D. Smith , K. Chandler , Y. Sadovsky , and D. M. Nelson . 2000 Apoptosis in human cultured trophoblasts is enhanced by hypoxia and diminished by epidermal growth factor. Am. J. Physiol. Cell Physiol. 278:C982–C988. 10794672 10.1152/ajpcell.2000.278.5.C982

[phy213049-bib-0025] Li, Z. , R. Zeki , L. Hilder , and E. A. Sullivan . 2012 Australia's mothers and babies 2010. AIHW National Perinatal Epidemiology and Statistics, Canberra.

[phy213049-bib-0026] Lie, S. , M. Hui , I. C. McMillen , B. S. Muhlhausler , G. S. Posterino , S. L. Dunn , et al. 2014 Exposure to rosiglitazone, a PPAR‐gamma agonist, in late gestation reduces the abundance of factors regulating cardiac metabolism and cardiomyocyte size in the sheep fetus. Am. J. Physiol. Regul. Integr. Comp. Physiol. 306:R429–R437. 24477540 10.1152/ajpregu.00431.2013 PMC3949109

[phy213049-bib-0027] Limesand, S. W. , P. J. Rozance , G. O. Zerbe , J. C. Hutton , and W. W. Jr Hay . 2006 Attenuated insulin release and storage in fetal sheep pancreatic islets with intrauterine growth restriction. Endocrinology 147:1488–1497. 16339204 10.1210/en.2005-0900

[phy213049-bib-0028] MacLaughlin, S. M. , S. K. Walker , C. T. Roberts , D. O. Kleemann , and I. C. McMillen . 2005 Periconceptional nutrition and the relationship between maternal body weight changes in the periconceptional period and feto‐placental growth in the sheep. J. Physiol. 565:111–124. 15774513 10.1113/jphysiol.2005.084996 PMC1464503

[phy213049-bib-0029] MacLaughlin, S. M. , S. K. Walker , D. O. Kleemann , J. P. Sibbons , D. N. Tosh , S. Gentili , et al. 2007 Impact of periconceptional undernutrition on adrenal growth and adrenal insulin‐like growth factor and steroidogenic enzyme expression in the sheep fetus during early pregnancy. Endocrinology 148:1911–1920. 17194740 10.1210/en.2006-0761

[phy213049-bib-0030] Mayhew, T. M. , C. Ohadike , P. N. Baker , I. P. Crocker , C. Mitchell , and S. S. Ong . 2003 Stereological investigation of placental morphology in pregnancies complicated by pre‐eclampsia with and without intrauterine growth restriction. Placenta 24:219–226. 12566249 10.1053/plac.2002.0900

[phy213049-bib-0031] McGillick, E. V. , J. L. Morrison , I. C. McMillen , and S. Orgeig . 2014 Intrafetal glucose infusion alters glucocorticoid signaling and reduces surfactant protein mRNA expression in the lung of the late‐gestation sheep fetus. Am. J. Physiol. Regul. Integr. Comp. Physiol. 307:R538–R545. 24990855 10.1152/ajpregu.00053.2014

[phy213049-bib-0032] McMillen, I. C. , and J. S. Robinson . 2005 Developmental origins of the metabolic syndrome: prediction, plasticity, and programming. Physiol. Rev. 85:571–633. 15788706 10.1152/physrev.00053.2003

[phy213049-bib-0033] McMillen, I. C. , M. B. Adams , J. T. Ross , C. L. Coulter , G. Simonetta , J. A. Owens , et al. 2001 Fetal growth restriction: adaptations and consequences. Reproduction 122:195–204. 11467970 10.1530/rep.0.1220195

[phy213049-bib-0034] Morrison, J. L. 2008 Sheep models of intrauterine growth restriction: fetal adaptations and consequences. Clin. Exp. Pharmacol. Physiol. 35:730–743. 18498533 10.1111/j.1440-1681.2008.04975.x

[phy213049-bib-0035] Morrison, J. L. , K. J. Botting , J. L. Dyer , S. J. Williams , K. L. Thornburg , and I. C. McMillen . 2007 Restriction of placental function alters heart development in the sheep fetus. Am. J. Physiol. Regul. Integr. Comp. Physiol. 293:R306–R313. 17428893 10.1152/ajpregu.00798.2006

[phy213049-bib-0036] Muhlhausler, B. S. , J. A. Duffield , S. E. Ozanne , C. Pilgrim , N. Turner , J. L. Morrison , et al. 2009 The transition from fetal growth restriction to accelerated postnatal growth: a potential role for insulin signalling in skeletal muscle. J. Physiol. 587:4199–4211. 19622603 10.1113/jphysiol.2009.173161 PMC2754360

[phy213049-bib-0037] Nelson, D. M. , S. D. Smith , T. C. Furesz , Y. Sadovsky , V. Ganapathy , C. A. Parvin , et al. 2003 Hypoxia reduces expression and function of system A amino acid transporters in cultured term human trophoblasts. Am. J. Physiol. Cell Physiol. 284:C310–C315. 12388062 10.1152/ajpcell.00253.2002

[phy213049-bib-0038] Owens, J. A. , J. Falconer , and J. S. Robinson . 1987a Effect of restriction of placental growth on fetal and utero‐placental metabolism. J. Dev. Physiol. 9:225–238. 3611639

[phy213049-bib-0039] Owens, J. A. , J. Falconer , and J. S. Robinson . 1987b Restriction of placental size in sheep enhances efficiency of placental transfer of antipyrine, 3‐O‐methyl‐D‐glucose but not of urea. J. Dev. Physiol. 9:457–464. 3693824

[phy213049-bib-0040] Passmore, M. , M. Nataatmadja , and J. F. Fraser . 2009 Selection of reference genes for normalisation of real‐time RT‐PCR in brain‐stem death injury in Ovis aries. BMC Mol. Biol. 10:72. 19624860 10.1186/1471-2199-10-72 PMC2721835

[phy213049-bib-0041] Poudel, R. , I. C. McMillen , S. L. Dunn , S. Zhang , and J. L . 2015 Morrison. Impact of chronic hypoxemia on blood flow to the brain, heart, and adrenal gland in the late‐gestation IUGR sheep fetus. Am. J. Physiol. Regul. Integr. Comp. Physiol. 308:R151–R162. 25427766 10.1152/ajpregu.00036.2014

[phy213049-bib-0042] Pringle, K. G. , K. L. Kind , J. G. Thompson , and C. T. Roberts . 2007 Complex interactions between hypoxia‐inducible factors, insulin‐like growth factor‐II and oxygen in early murine trophoblasts. Placenta 28:1147–1157. 17658597 10.1016/j.placenta.2007.05.009

[phy213049-bib-0043] Regnault, T. R. , R. J. Orbus , B. de Vrijer , M. L. Davidsen , H. L. Galan , R. B. Wilkening , et al. 2002 Placental expression of VEGF, PlGF and their receptors in a model of placental insufficiency‐intrauterine growth restriction (PI‐IUGR). Placenta 23:132–144. 11945079 10.1053/plac.2001.0757

[phy213049-bib-0044] Regnault, T. R. H. , B. de Vrijer , H. L. Galan , M. L. Davidsen , K. A. Trembler , F. C. Battaglia , et al. 2003 The relationship between transplacental O2 diffusion and placental expression of PIGF, VEGF and their receptor in a placental insufficiency model of fetal growth restriction. J. Physiol. 550:641–656. 12740423 10.1113/jphysiol.2003.039511 PMC2343042

[phy213049-bib-0045] Regnault, T. R. , A. M. Marconi , C. H. Smith , J. D. Glazier , D. A. Novak , C. P. Sibley , et al. 2005 Placental amino acid transport systems and fetal growth restriction–a workshop report. Placenta 26(Suppl A):S76–S80. 15837072 10.1016/j.placenta.2005.02.006

[phy213049-bib-0046] Regnault, T. R. H. , B. de Vrijer , H. L. Galan , R. B. Wilkening , F. C. Battaglia , and G. Meschia . 2007 Development and mechanisms of fetal hypoxia in severe fetal growth restriction. Placenta 28:714–723. 16962658 10.1016/j.placenta.2006.06.007

[phy213049-bib-0047] Roberts, C. T. , A. Sohlstrom , K. L. Kind , R. A. Earl , T. Y. Khong , J. S. Robinson , et al. 2001 Maternal food restriction reduces the exchange surface area and increases the barrier thickness of the placenta in the guinea‐pig. Placenta 22:177–185. 11170822 10.1053/plac.2000.0602

[phy213049-bib-0048] Robinson, J. , S. Chidzanja , K. Kind , F. Lok , P. Owens , and J. Owens . 1995 Placental control of fetal growth. Reprod. Fertil. Dev. 7:333–344. 8606942 10.1071/rd9950333

[phy213049-bib-0049] Robinson, J. S. , K. M. Hartwich , S. K. Walker , J. J. Erwich , and J. A. Owens . 1997 Early influences on embryonic and placental growth. Acta Paediatr. (Oslo, Norway: 1992) Suppl. 423:159–163; discussion 164. 10.1111/j.1651-2227.1997.tb18401.x 9401564

[phy213049-bib-0050] Sferruzzi‐Perri, A. N. , J. A. Owens , P. Standen , and C. T. Roberts . 2008 Maternal insulin‐like growth factor‐II promotes placental functional development via the type 2 IGF receptor in the guinea pig. Placenta 29:347–355. 18339421 10.1016/j.placenta.2008.01.009

[phy213049-bib-0051] Shams, M. , M. D. Kilby , D. A. Somerset , A. J. Howie , A. Gupta , P. J. Wood , et al. 1998 11Beta‐hydroxysteroid dehydrogenase type 2 in human pregnancy and reduced expression in intrauterine growth restriction. Hum. Reprod. (Oxford, England) 13:799–804. 10.1093/humrep/13.4.799 9619527

[phy213049-bib-0052] Smith, S. C. , P. N. Baker , and E. M. Symonds . 1997 Increased placental apoptosis in intrauterine growth restriction. Am. J. Obstet. Gynecol. 177:1395–1401. 9423741 10.1016/s0002-9378(97)70081-4

[phy213049-bib-0053] Soo, P. S. , J. Hiscock , K. J. Botting , C. T. Roberts , A. K. Davey , and J. L. M . 2012 Maternal undernutrition reduces P‐glycoprotein in guinea pigs placenta and developing brain in late gestation. Reprod. Toxicol. 33:374–381. 22326852 10.1016/j.reprotox.2012.01.013

[phy213049-bib-0054] Vatnick, I. , P. A. Schoknecht , R. Darrigrand , and A. W. Bell . 1991 Growth and metabolism of the placenta after unilateral fetectomy in twin pregnant ewes. J. Dev. Physiol. 15:351–356. 1753075

[phy213049-bib-0055] Vonnahme, K. A. , W. J. Arndt , M. L. Johnson , P. P. Borowicz , and L. P. Reynolds . 2008 Effect of morphology on placentome size, vascularity, and vasoreactivity in late pregnant sheep. Biol. Reprod. 79:976–982. 18685124 10.1095/biolreprod.108.070748

[phy213049-bib-0056] de Vrijer, B. , M. L. Davidsen , R. B. Wilkening , R. V. Anthony , and T. R. Regnault . 2006 Altered placental and fetal expression of IGFs and IGF‐binding proteins associated with intrauterine growth restriction in fetal sheep during early and mid‐pregnancy. Pediatr. Res. 60:507–512. 16966353 10.1203/01.PDR.0000242364.78002.71

[phy213049-bib-0057] Wang, K. C. , L. Zhang , I. C. McMillen , K. J. Botting , J. A. Duffield , S. Zhang , et al. 2011 Fetal growth restriction and the programming of heart growth and cardiac insulin‐like growth factor 2 expression in the lamb. J. Physiol. 589:4709–4722. 21807611 10.1113/jphysiol.2011.211185 PMC3213418

[phy213049-bib-0058] Wang, K. C. , C. H. Lim , I. C. McMillen , J. A. Duffield , D. A. Brooks , and J. L. Morrison . 2013 Alteration of cardiac glucose metabolism in association to low birth weight: experimental evidence in lambs with left ventricular hypertrophy. Metabolism 62:1662–1672. 23928106 10.1016/j.metabol.2013.06.013

[phy213049-bib-0059] Ward, J. W. , A. J. Forhead , F. B. Wooding , and A. L. Fowden . 2006 Functional significance and cortisol dependence of the gross morphology of ovine placentomes during late gestation. Biol. Reprod. 74:137–145. 16177219 10.1095/biolreprod.105.046342

[phy213049-bib-0060] Zamudio, S. , M. U. Baumann , and N. P. Illsley . 2006 Effects of chronic hypoxia in vivo on the expression of human placental glucose transporter. Placenta 27:49–55. 16310037 10.1016/j.placenta.2004.12.010 PMC4497571

[phy213049-bib-0061] Zhang, S. , L. Rattanatray , S. M. MacLaughlin , J. E. Cropley , C. M. Suter , L. Molloy , et al. 2010 Periconceptional undernutrition in normal and overweight ewes leads to increased adrenal growth and epigenetic changes in adrenal IGF2/H19 gene in offspring. FASEB J. 24:2772–2782. 20371620 10.1096/fj.09-154294

[phy213049-bib-0062] Zhang, S. , T. R. Regnault , P. L. Barker , K. J. Botting , I. C. McMillen , C. M. McMillan , et al. 2015 Placental adaptations in growth restriction. Nutrients 7:360–389. 25580812 10.3390/nu7010360 PMC4303845

